# Coarse snapshots of oxygen-dissociation intermediates of a giant hemoglobin elucidated by determining the oxygen saturation in individual subunits in the crystalline state

**DOI:** 10.1107/S2052252521009386

**Published:** 2021-09-30

**Authors:** Nobutaka Numoto, Yoshiaki Kawano, Hideo Okumura, Seiki Baba, Yoshihiro Fukumori, Kunio Miki, Nobutoshi Ito

**Affiliations:** a Medical Research Institute, Tokyo Medical and Dental University (TMDU), 1-5-45 Yushima, Bunkyo-ku, Tokyo 113-8510, Japan; b RIKEN SPring-8 Center, 1-1-1 Kouto, Sayo-cho, Sayo-gun, Hyogo 679-5148, Japan; c Protein Crystal Analysis Division, Japan Synchrotron Radiation Research Institute, 1-1-1 Kouto, Sayo-cho, Sayo-gun, Hyogo 679-5198, Japan; d Nano Life Science Institute, Kanazawa University, Kanazawa, Ishikawa 920-1192, Japan; e Graduate School of Science, Kyoto University, Sakyo-ku, Kyoto 606-8502, Japan

**Keywords:** giant hemoglobin, allosteric transition intermediates, oxygen saturation

## Abstract

Cooperative oxygen binding of hemoglobin (Hb) has been studied for over half a century as a representative example of the allostericity of proteins. The most important problem remaining to be solved is the lack of structural information on the intermediates between the oxygenated and deoxygenated forms. In order to characterize the intermediate structures, it is necessary to obtain intermediate-state crystals, determine their oxygen saturations and then determine the oxygen saturations of each of their constituent subunits, all of which are challenging issues even now. Here, intermediate forms of the 400 kDa giant Hb from the tubeworm *Oligobrachia mashikoi* are reported. To overcome the above problems without any artificial modifications to the protein or prosthetic groups, intermediate crystals of the giant Hb were prepared from fully oxygenated crystals by a soaking method. The oxygen saturation of the crystals was measured by *in situ* observation with a microspectrophotometer using thin plate crystals processed by an ultraviolet laser to avoid saturation of absorption. The oxygen saturation of each subunit was determined by occupancy refinement of the bound oxygen based on ambient temperature factors. The obtained structures reveal the detailed relationship between the structural transition and oxygen dissociation. The dimer subassembly of the giant Hb shows strong correlation with the local structural changes at the heme pockets. Although some local ternary-structural changes occur in the early stages of the structural transition, the associated global ternary-structural and quaternary-structural changes might arise at about 50% oxygen saturation. The models based on coarse snapshots of the allosteric transition support the conventional two-state model of Hbs and provide the missing pieces of the intermediate structures that are required for full understanding of the allosteric nature of Hbs in detail.

## Abstract 2

Crystal structures of oxy–deoxy intermediates of a 400 kDa giant hemoglobin reveal the allosteric transition intermediates of the unmodified protein and unrestrained oxygen saturation, which have been missing pieces in the study of the allosteric behavior of hemoglobins over the last half century.

## 1. Introduction   

The mechanism of cooperative oxygen binding of hemoglobin (Hb) was explained about half a century ago by Perutz’s stereochemical model (Perutz, 1970 ▸) based on the crystal structures of oxygenated (oxy) Hb and deoxygenated (deoxy) Hb and the theoretical Monod–Wyman–Changeux (MWC) model (Monod *et al.*, 1965 ▸) for multi-subunit allosteric proteins. However, a comprehensive understanding of the allosteric mechanism of Hb has not been achieved. The most important information that is still missing comprises the intermediate structures between the oxy and deoxy states.

Several intermediate structures of human Hb, in which the tetrameric Hb is artificially half-CO-liganded and cross-linked (Shibayama, 2020 ▸; Shibayama *et al.*, 2014 ▸, 2017 ▸, 2020 ▸), have been reported. A series of large conformational changes were reported in detail, but because the ligand saturation rate was artificially restrained, there is still room for exploration with regard to how the structural changes occur at other ligand-saturation rates. Another example of the intermediate structure has been reported for an invertebrate homodimeric Hb from the clam *Scapharca inaequivalvis* (Knapp *et al.*, 2006 ▸, 2009 ▸; Nienhaus *et al.*, 2007 ▸). The ligand saturation was changed continuously and reversibly in the crystalline state (Knapp *et al.*, 2006 ▸); although no large quaternary-structural change was observed, small changes in ternary structure and hydrogen-bond networks at the interface of the dimer structure were thought to play an essential role in the cooperative mechanism.

In order to analyze the ‘intact’ oxy–deoxy intermediate involving significant quaternary-structural change, it is necessary to overcome some technical problems. Firstly, crystals with an arbitrary oxygen-bound fraction must be produced. Secondly, the oxygen-bound fraction of the crystals (*i.e.* the average fraction for the whole subunits) used in the diffraction experiment must be accurately measured. Finally, the oxygen-bound fraction must be determined for each subunit.

In this study, to overcome these problems, we analyzed the 400 kDa giant Hb from the tubeworm *Oligobrachia mashikoi* (*Oli*Hb; Nakagawa *et al.*, 2005 ▸; Numoto *et al.*, 2005 ▸). We considered that its high oxygen affinity, moderate cooperativity (*P*_50_ and *n*_max_ of 0.19 mmHg and 2.5, respectively; Aki *et al.*, 2007 ▸) and significant ternary- and quaternary-structural changes (Numoto *et al.*, 2008*a* ▸,*b* ▸, 2014 ▸) would be suitable for analysis of the intermediate structures. We used our previously reported soaking method (Numoto *et al.*, 2014 ▸) to gradually release the bound oxygen from oxy crystals of *Oli*Hb, so that crystals with arbitrary oxygen saturation could be prepared. The oxygen saturation fraction of the crystals was measured by *in situ* observation using an online microspectrophotometer at SPring-8. To avoid saturation of absorption, the crystals were processed into thin plates using a laser. The oxygen saturation (*i.e.* occupancy) of each subunit was determined based on the ambient temperature factors.

The structures revealed that local ternary-structural changes are induced at an early stage of oxygen dissociation, and subsequent global ternary-structural changes and large quaternary-structural rearrangement might arise when about half of the oxygen molecules have dissociated from all of the subunits. Our structures should provide a more accurate understanding of the allosteric mechanism of Hbs.

## 2. Methods   

### 2.1. Sample preparation and crystallization   

The tubeworm *O. mashikoi* was collected as described previously (Sasayama *et al.*, 2003 ▸). The 400 kDa Hb was purified as reported previously (Nakagawa *et al.*, 2005 ▸; Numoto *et al.*, 2008*b* ▸). Briefly, the blood of the worm was loaded onto a size-exclusion column (Sephacryl S-300 HR; Cytiva), and the fractions with a ratio of absorbance above 2.5 at 410/280 nm were collected. Subsequent purification was performed using a hydrophobic interaction column (Resource 15 PHE; GE Healthcare). The protein was desalted and concentrated to 30 mg ml^−1^ by ultrafiltration in 50 m*M* Tris–HCl pH 7.4.

Crystals were obtained by the sitting-drop vapor-diffusion method at 20°C using equal volumes of protein solution and reservoir solution consisting of 11–12%(*w*/*v*) polyethylene glycol (PEG) 10 000, 10 m*M* CaCl_2_, 200 m*M* Tris–HCl pH 8.0. Crystals grew to typical dimensions of 200 × 200 × 400 µm.

### 2.2. Deoxygenation of the oxy crystals   

The obtained crystals appeared slightly brownish, indicating that they contained a small amount of the oxidized met form (Numoto *et al.*, 2008*b* ▸). To reduce the met form and convert the crystals to the complete oxy form, the crystals were soaked in buffer consisting of 10 m*M* ascorbic acid, 50 m*M* CaCl_2_, 12% PEG 10 000, 200 m*M* Tris–HCl pH 8.0 for 10 min. The crystals were then soaked in buffer consisting of 50 m*M* sodium hydrosulfite, 20%(*v*/*v*) PEG 400, 12% PEG 10 000, 200 m*M* Tris–HCl pH 8.0. The concentrations of sodium hydrosulfite and PEG 400 were increased in a stepwise manner (Numoto *et al.*, 2014 ▸) in three steps and the crystals were then incubated for 10–90 s. The crystals were immediately flash-cooled under a nitrogen-gas stream at −183°C.

### 2.3. Crystal processing and microspectrometry   

The cooled crystals were processed in a crystal-processing machine using an ultraviolet laser (Basu *et al.*, 2019 ▸) under a nitrogen-gas cryostream. The absorption spectra of the processed crystals were measured and corrected for the air blank baseline under a nitrogen-gas cryostream using the online microspectrophotometer at beamline BL38B1 (Shimizu *et al.*, 2013 ▸) or offline equipment (Chiu *et al.*, 2006 ▸) developed at SPring-8.

### 2.4. Data collection, model building and refinement   

X-ray diffraction experiments were performed on beamlines BL38B1 at SPring-8 and BL-1A, BL-5A and BL-17A at the KEK Photon Factory. To reduce radiation damage, data were collected from multiple exposure points or using a helical scan procedure. All data were processed and scaled using *XDS* (Kabsch, 2010 ▸) and truncated using the *CCP*4 software suite (Winn *et al.*, 2011 ▸). The structures of *Oli*Hb (PDB entries 2zs0 and 2zfo; Numoto *et al.*, 2008*a* ▸,*b* ▸) were used as search models to determine the initial phases by the molecular-replacement method using *Phaser* (McCoy *et al.*, 2007 ▸). Several cycles of manual model rebuilding and refinement were performed using *Coot* (Emsley *et al.*, 2010 ▸) and *Phenix* (Liebschner *et al.*, 2019 ▸), respectively.

The occupancies of the bound oxygen molecules in each subunit were estimated as follows. The refinements were performed for the model omitting oxygen, but including all other prosthetic groups and solvent molecules. When the refinement was stable, oxygen molecules were added to the model with the same geometry as in the previously reported high-resolution structure of the oxy form of *Oli*Hb. The distance between the Fe atom of the heme and the O1 atom of the oxygen molecule was strongly restrained to the equivalent distance in the corresponding oxy form. Refinements of *xyz* coordinates, individual atomic displacement parameters (*B* factors) and group occupancies for the oxygen molecules of each subunit were performed, but all parameters for the other atoms were fixed. The refined occupancies of the oxygen molecules were manually adjusted, and the individual *B* factors were refined. Iterative occupancy adjustments and *B*-factor refinements of the oxygen molecule were carried out until the refined *B* factors of the oxygen molecules converged within the standard deviation of the refined *B* factors of the surrounding atoms (Supplementary Table S1). Because the resolutions of our data are not high enough to model the protein portion as a mixture of conformations such as oxy and deoxy forms, and we have no confirmation of the oxy:deoxy ratio of the protein structures by X-ray-independent data, we modeled the protein and heme portion as a single conformer, with the exception of a few residues. Tight restraints to maintain distances between the N atom of the proximal His (F8) and the Fe atom of heme, and between the oxygen molecule and the Fe atom of heme, were introduced. The final models were validated by *MolProbity* (Chen *et al.*, 2010 ▸). The statistics for data collection and refinement are summarized in Table 1 ▸. The figures were prepared using *PyMOL* (http://www.pymol.org/).

## 3. Results   

### 3.1. Detection of oxygen dissociation in crystalline *Oli*Hb   

**Figure 1:**
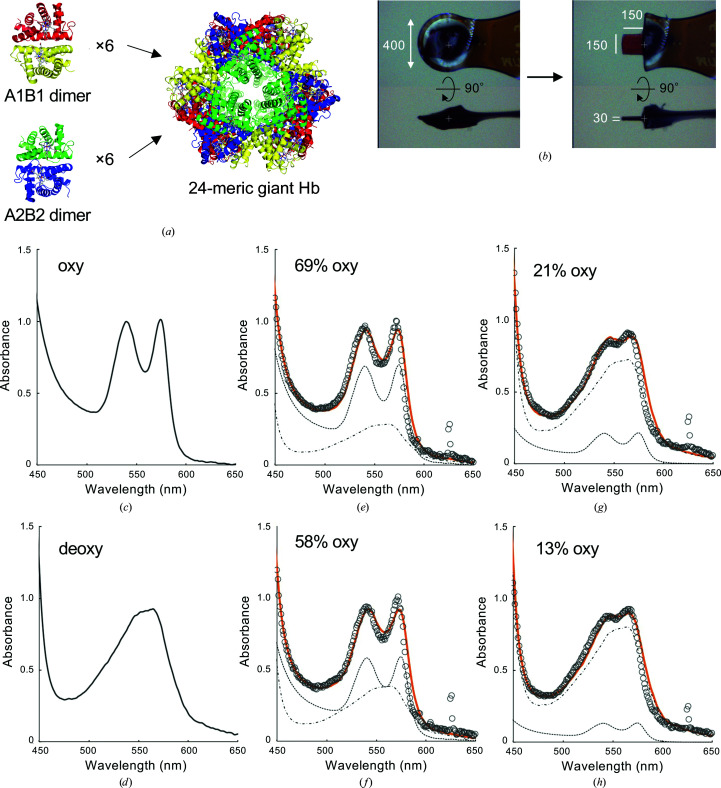
Determination of the oxygen saturation fraction of the crystals. (*a*) Ribbon diagram of *Oli*Hb. The A1, A2, B1 and B2 subunits are shown in red, green, yellow and blue, respectively. The A1 and B1 subunits form a dimer subassembly, and the A2 and B2 subunits form a similar dimer subassembly. Six A1B1 dimers and six A2B2 dimers form a spherical 24-mer assembly as a biological unit that is directly dissolved in the blood of the worm. (*b*) Crystal processing via ultraviolet laser. The image on the left is the crystal before processing, mounted in the cryo-stream. The image on the right is the crystal after processing. The values by the white lines are lengths in µm. (*c*, *d*) Reference absorption spectra of the oxy and deoxy forms obtained for *Oli*Hb in solution are shown as solid black lines. (*e*–*h*) The black circles are the absorptions measured from the processed crystals using a microspectrophotometer, and the orange lines are the fits calculated by a least-squares fit of the observed absorption spectrum to a linear combination of the oxy and deoxy reference spectra. The component reference spectra are shown as black dashed lines. Peaks around 630 nm are derived from the emission lines of the Hg–Xe lamp.

A previous study demonstrated that an oxy crystal of the giant Hb from another annelid, *Lammelibrachia satsuma*, can be made to shift to the deoxy form while maintaining the crystalline state by soaking in a solution containing PEG 400 and 50 m*M* sodium hydrosulfite (Numoto *et al.*, 2014 ▸). The same method was successfully applied to *Oli*Hb [Fig. 1 ▸(*a*)]. To obtain crystals of the oxy–deoxy intermediate state, we tested various soaking times from 10 to 90 s followed by immediate flash-cooling under a nitrogen-gas stream at −178°C. The oxygenation state of the crystals was verified by the absorption spectra (450–700 nm) of the crystals using the online microspectrophotometer on beamline BL38B1 or an offline microspectrophotometer at SPring-8. For the microspectroscopy of protein crystals, very thin plate-like crystals are usually required to avoid signal saturation. In our case, a thickness of about 30 µm was most suitable to obtain a sufficient signal without signal saturation. Because it is very difficult to intentionally obtain protein crystals with a specific thickness, we trimmed crystals of various sizes to a plate-like shape with 30 µm thickness using a protein crystal-processing machine (Basu *et al.*, 2019 ▸) with an ultraviolet laser. After soaking and cooling, the crystals were processed by a laser to about 100 × 100 to 200 × 200 µm (slightly larger than the beam size of the microspectrophotometer) and 30 µm thickness under the cryo-stream conditions [Fig. 1 ▸(*b*)].

The spectra clearly showed patterns intermediate between oxy and deoxy states [Figs. 1 ▸(*c*)–1 ▸(*h*)]. The oxygen saturation fraction of the crystals was calculated by fitting a linear combination of the reference absorption spectra for the oxy and deoxy states. The observed oxygen saturation of the processed crystals was 100–50% in most cases, and few crystals with under 50% oxygen saturation could be obtained. This was probably due to the fact that the processed crystals used for observation were originally located in the center portion of the crystals and therefore would have been less susceptible to the soaking method. To obtain low oxygen saturation crystals, we first cooled and processed crystals into a thin plate shape, and then soaked the crystals in the solution for oxygen dissociation for several seconds and recooled them for observation. This pre-processing procedure resulted in a low oxygen saturation of around 20%. Finally, we obtained four crystals with 69%, 58%, 21% and 13% oxygen saturation, designated Crystals 1, 2, 3 and 4, respectively. Crystals 1 and 2 were prepared by the pre-soaking procedure, whereas Crystals 3 and 4 were prepared by the pre-processing procedure. These crystals were subjected to further data collection and structural analyses.

### 3.2. Determination of the oxygen saturation for each subunit via temperature factor   

**Figure 2:**
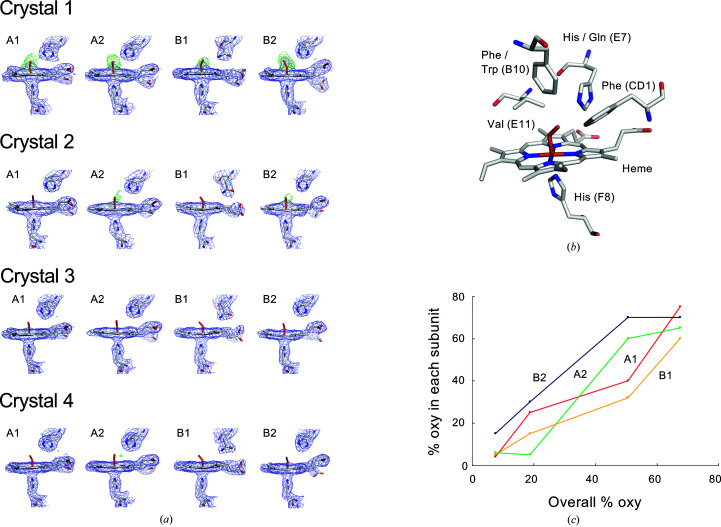
Determination of the oxygen saturation fraction of each subunit. (*a*) Electron densities superposed on the ligand sites of each subunit. The 2*F*_o_ − *F*_c_ maps (blue, contoured at 1.0σ) and the oxygen-omitted *F*_o_ − *F*_c_ maps (green, contoured at 2.5σ) are represented with stick models of the heme, oxygen, proximal histidine and distal histidine. (*b*) The residues to which the atoms subjected to the *B*-factor analysis belong are shown as stick models. (*c*) Transition of oxygen saturation in each subunit (vertical axis). The overall oxygen saturation of the crystals (horizontal axis) was determined as in Fig. 1 ▸.

The structures of Crystals 1, 2, 3 and 4 of *Oli*Hb were determined at 2.4, 2.7, 2.2 and 2.1 Å resolution, respectively. The observed electron densities for the bound oxygen molecules were different for each subunit [Fig. 2 ▸(*a*)]. Since the oxygen saturation determined by microspectroscopy is the average of all subunits, we need to estimate the oxygen saturation of each subunit in another way. Although the intensity of the difference Fourier map calculated from the oxygen-omitted model should reflect the oxygen saturation, quantification of the oxygen saturation (*i.e.* occupancy) using the difference Fourier map, such as by determining the sigma value, would seem to be difficult in practice. In fact, the electron density of the difference Fourier map of crystals with less than 50% saturation is too weak to be observed [Fig. 2 ▸(*a*)]. It is recognized that refinement of the occupancy by the usual method is unstable at low or medium resolutions. Therefore, we attempted to estimate the oxygen saturation of each subunit via refined atomic displacement parameters (*B* factors).

The *B* factor of each atom in the crystal structure should be similar to those of the atoms bonded to it. Therefore, the oxygen molecule, the Fe atom of the heme to which it is coordinated and the N^ɛ^ atom of the distal histidine to which it forms a hydrogen bond should exhibit similar *B* factors. We first refined an oxygen-omitted model and investigated the distribution of the refined *B* factors of the iron of the heme and the N^ɛ^ atom of the distal histidine together with other iron-coordinated atoms and the atoms of oxygen-surrounding residues [Fig. 2 ▸(*b*) and Supplementary Table S1]. We then determined the coarse occupancy, so that the refined *B* factors of oxygen matched the distribution of those of nearby atoms (Supplementary Table S1; see Section 2 for details). The occupancies of oxygen molecules in each subunit, as obtained by this method, agree with the observed difference Fourier map of the oxygen-omitted model, and notably the average of the occupancies of each subunit (*i.e.* the total oxygen saturation in the crystal) is roughly confirmed by the oxygen saturation determined via microspectroscopy. The average occupancies determined by the *B*-factor analysis (68%, 51%, 19% and 7.5%) turned out to be slightly lower than the oxygen saturation determined by microspectroscopic analysis for all four crystals. These facts may indicate that there was slight deoxygenation due to X-ray radiation during data collection, as reported for many heme proteins (Beitlich *et al.*, 2007 ▸; Pfanzagl *et al.*, 2020 ▸). The changes in the occupancy in each subunit [Fig. 2 ▸(*c*)] clearly reveal that those of the A1 and B1 subunits are strongly correlated with each other and that those of the A2 and B2 subunits are also strongly correlated with each other. Thus, there is a strong correlation between the subunits that form the dimer subassembly, as expected from previous structural studies (Numoto *et al.*, 2008*a* ▸,*b* ▸).

### 3.3. Ternary-structural change in each subunit   

**Figure 3:**
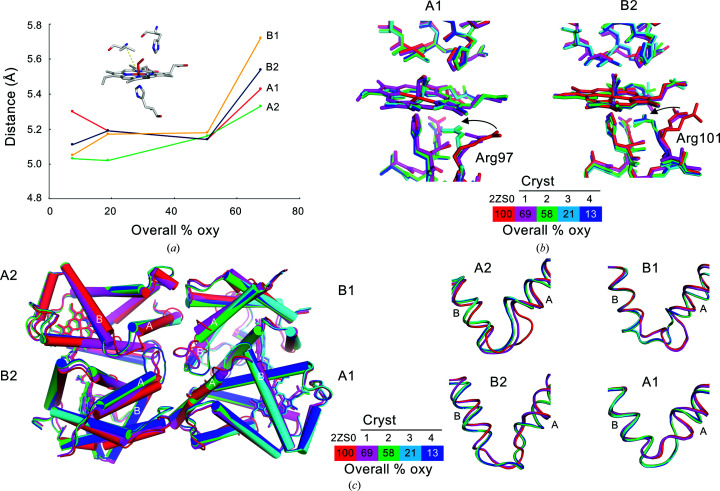
Ternary-structural transitions in each subunit. (*a*) Changes in the distance between the C^β^ atom of Val E11 and the iron of the heme (yellow dashed line in the inset) for each subunit. The overall oxygen saturation of the crystals (horizontal axis) was determined as in Fig. 1 ▸. (*b*) Superpositions around the heme of the A1 (left) and B2 (right) subunits. The stick models of fully oxygenated *Oli*Hb (PDB entry 2zs0; Numoto *et al.*, 2008*a* ▸) and Crystals 1, 2, 3 and 4 are drawn in red, magenta, green, cyan and blue, respectively. The conformational changes in A1 Arg97 and B2 Arg101 are indicated by black arrows. (*c*) Superpositions of the tetrameric subassemblies. The color schemes are the same as in (*b*). The A and B helices of each subunit are labeled. Close-up views of the superpositions of the AB loops are shown in the right panels.

We have previously reported the ternary- and quaternary-structural changes between oxy and deoxy forms in giant Hbs from annelids. The most remarkable ternary change around the heme pocket is the protrusion of Val E11 in each subunit towards the heme pocket in the deoxy (unliganded) structure (Numoto *et al.*, 2008*b* ▸, 2014 ▸). In the present study, to evaluate the structural transition at the heme pocket, we traced the distances between the C^β^ atom of Val E11 and the iron of the heme [Fig. 3 ▸(*a*)]. The results clearly indicate that the structural change at the heme pocket is not a gradual process but rather a single-step transition in all of the subunits. Notably, structural changes occur in the A2 and B2 subunits even though the oxygen occupancy remains over 50%. Similar single-step transitions are observed in the traces of the distances between the N^ɛ^ atom of His E7 (distal His) and the iron of the heme (Supplementary Fig. S1), where His E7 moves away from the heme as oxygen dissociates, so that a hydrogen bond between His E7 and the oxygen molecule is disrupted. These results strongly support the MWC two-state model (Monod *et al.*, 1965 ▸) for the explanation of the allosteric nature of Hbs. This model is further supported by other conformational changes observed at Arg97 of the A1 subunit and Arg101 of the B2 subunit [Fig. 3 ▸(*b*)], located +3 from the proximal His (F8). The side chains of these arginine residues are oriented towards the solvent region in Crystal 1, whereas in Crystals 2, 3 and 4 their orientations are shifted towards the space between His F8 and the heme. The bulky guanidino group would affect the coordination geometry between the heme iron and the proximal His F8, and may reduce the oxygen-binding affinity of the heme.

On the other hand, structural changes deviating from the MWC two-state model are also observed. Analyses of the interatomic distance for combinations of atoms of the oxygen-surrounding residues (Supplementary Fig. S1) demonstrate that not all residues undergo a single-step structural transition. Besides the structural transitions around the heme pockets, the largest ternary-structural changes between the oxy and deoxy form in each subunit are observed in the AB loop region. In Crystal 1, the conformation of the AB loop of the A2 subunit matches that of the deoxy form, whereas the AB loops of the remaining three subunits shows the conformation of the oxy form [Fig. 3 ▸(*c*)]. In Crystals 2, 3 and 4 all of the AB loops show the deoxy conformation. These facts strongly suggest that the AB loop of the A2 subunit is most flexible and most readily transits from the oxy to the deoxy form in the early stages of oxygen dissociation. Indeed, the structural change in the AB loop of the A2 subunit is smaller than those in the AB loops at the other subunits, and the structure of the AB loop of the A2 subunit is also more flexible (*i.e.* it has the weakest electron density) than the other AB loops. After the structural transitions of the AB loop of the A2 subunit is completed, the structural transitions in all of the AB loops are considered to be completed at an average of 58% oxygen saturation.

### 3.4. Quaternary-structural change   

**Figure 4:**
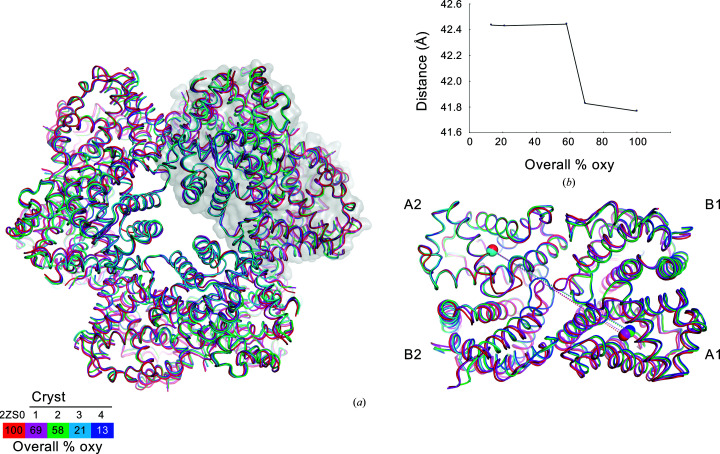
Quaternary-structural transitions in the overall structure. (*a*) Superposition of the 24-meric assemblies of *Oli*Hb. The color schemes are the same as in Fig. 3 ▸. A tetrameric subassembly is represented as a transparent surface model superposed on the overall structures, and a close-up view is shown in the right panel. The centers of gravity of each A1 and A2 subunit are represented as spheres of the same color. (*b*) Transition of the distance between the centers of gravity of the A1 and A2 subunits [dashed line in (*a*)].

Consistent with the ternary-structural changes around the heme pockets, the quaternary structure of Crystal 1 matches that of the oxy form and the quaternary structures of Crystals 2, 3 and 4 match that of the deoxy form [Fig. 4 ▸(*a*)]. Analysis of the relative distance between the coordinates of the centers of gravity of the A1 and A2 subunits [Fig. 4 ▸(*b*)] reveals that the quaternary change of *Oli*Hb is a single-step transition, as observed for the ternary transition at Val E11. It is strongly suggested that the structural change to the deoxy form is almost completed at 20% oxygen saturation or less, because there is almost no difference between the structures of Crystals 3 and 4 (r.m.s.d. of 0.148 Å for all residues). Thus, the quaternary transition is completed in synchronization with the completion of the ternary-structural change of the AB loops and also that of Val E11 in the heme pocket of each subunit.

## 4. Discussion   

The crystal structures reported in this study reveal the oxy–deoxy intermediates of *Oli*Hb, providing coarse snapshots of the allosteric transition of this multimeric Hb. In addition to the ternary- and quaternary-structural rearrangements, the oxygen saturation of each subunit was determined via careful refinements guided by the temperature factor. Therefore, the structures in this study elucidate the ‘intact’ oxy–deoxy intermediates without any mutation or artificial modification of the protein and without the use of ligand analogs such as carbon monoxide.

The continuous studies on human tetrameric Hb by Shibayama and coworkers have revealed a novel intermediate conformer designated TR (Shibayama *et al.*, 2014 ▸) which exhibits a structure and oxygen affinity intermediate between those of the canonical T and R states of Hb. There is no significant quaternary intermediate structure in our snapshots, yet local ternary-structural changes are observed in the early stage of the oxygen dissociation process. Analysis with finer snapshots may provide an intermediate quaternary structure of *Oli*Hb.

In the structure of our Crystal 2, the A1B1 dimer showed lower oxygen saturation than the A2B2 dimer, although both dimers exhibit almost equivalent ternary and quaternary structures to those of the deoxy structure. Therefore, it was strongly suggested that the oxygen affinity would be determined not only by the ternary and quaternary structures themselves, but also by other factors. One possible determinative factor is the structural dynamics. Several studies have attempted to explain the allosteric nature of Hb (for example the global allostery model of Yonetani & Laberge, 2008 ▸) or the allosteric nature of other proteins (Motlagh *et al.*, 2014 ▸) by incorporating structural dynamics. Our coarse snapshots of the oxy–deoxy intermediates of this giant Hb will provide useful structural information for the verification of allosteric models by structural dynamics, which are often studied by molecular-dynamics simulations or other computational analyses.

In conclusion, the structures in this study provide allosteric transition intermediates for a giant Hb. Unmodified and unrestrained oxygen saturation samples reveal missing pieces of the structural information of the allosteric intermediates of Hb. Although our models largely support the conventional MWC two-state model for the allosteric nature of Hbs, room remains for more detailed understanding by investigation of the structural dynamics.

## Supplementary Material

PDB reference: oxy–deoxy intermediate of 400 kDa giant hemoglobin at 69% oxygen saturation, 7e96

PDB reference: at 21% oxygen saturation, 7e98

PDB reference: at 58% oxygen saturation, 7e97

PDB reference: at 13% oxygen saturation, 7e99

Supplementary Table and Figure. DOI: 10.1107/S2052252521009386/lz5049sup1.pdf

## Figures and Tables

**Table 1:** Data-collection and refinement statistics Values in parentheses are for the highest resolution shell.

Crystal (PDB code)	Crystal 1 (7e96)	Crystal 2 (7e97)	Crystal 3 (7e98)	Crystal 4 (7e99)
Data collection				
Preparation	Pre-soaking	Pre-soaking	Pre-processed	Pre-processed
Wavelength (Å)	0.9800	0.9800	1.1000	1.1000
Space group	*H*32	*H*32	*H*32	*H*32
*a*, *c* (Å)	110.2, 274.7	111.0, 272.4	112.2, 271.4	110.9, 271.0
Resolution (Å)	50–2.40 (2.54–2.40)	50–2.70 (2.87–2.70)	50–2.20 (2.33–2.20)	50–2.10 (2.23–2.10)
No. of observations	255607	176233	347797	380447
No. of unique reflections	25540	18081	33753	37707
Completeness (%)	100 (99.9)	99.8 (99.2)	99.7 (98.7)	99.8 (99.1)
Average *I*/σ(*I*)	12.6 (1.7)	9.0 (1.5)	10.4 (1.5)	11.0 (1.7)
Multiplicity	10.0 (10.3)	9.7 (9.4)	10.3 (10.6)	10.1 (10.1)
*R*_merge_ (%)	13.1 (129)	20.8 (137)	19.0 (190)	17.8 (156)
CC_1/2_ (%)	99.8 (59.6)	99.8 (99.2)	99.7 (66.6)	99.7 (77.9)
Refinement				
*R*/*R*_free_ (%)	19.2/23.9	20.6/25.2	18.8/21.9	18.1/22.0
Average *B* factor (Å^2^)	52.3	61.2	39.6	36.5
No. of atoms				
Protein	4271	4263	4265	4263
Heme and oxygen	180	180	180	180
Ca^2+^	1	—	—	—
Glycerol	—	—	6	—
Water	155	17	338	393
R.m.s.d. from ideal				
Bond lengths (Å)	0.007	0.003	0.003	0.003
Angles (°)	0.85	0.63	0.67	0.71
Ramachandran plot				
Favored region (%)	98.1	97.2	98.4	98.0
Allowed region (%)	1.9	2.8	1.4	2.0
Outlier region (%)	0	0	0.2	0
