# Supplementary material for: Coarse snapshots of oxygen-dissociation intermediates of a giant hemoglobin elucidated by determining the oxygen saturation in individual subunits in the crystalline state
Source: IUCrJ. 2021 Sep 30;8(Pt 6):954–62. doi: 10.1107/S2052252521009386 (PMC8562662; doi:10.1107/S2052252521009386)
Supplement: Supplementary file 1 [file m-08-00954-sup1.pdf]

**IUCrJ**

**Volume 8 (2021)**

**Supporting information for article:**

# Coarse snapshots of oxygen-dissociation intermediates of a giant hemoglobin elucidated by determining the oxygen saturation in individual subunits in the crystalline state

**Nobutaka Numoto, Yoshiaki Kawano, Hideo Okumura, Seiki Baba, Yoshihiro Fukumori, Kunio Miki and Nobutoshi Ito**

**Table S1** Refined *B*-factors and occupancies of the atoms of oxygen-surrounding residues.

| Cryst 1 (69% oxy) | | | | Cryst 2 (58% oxy) | | | |
|---|---|---|---|---|---|---|---|
| A1 | | | | A1 | | | |
| Residue | atom | *B*-factor | Occ. | Residue | atom | *B*-factor | Occ. |
| Heme | FE | 51.23 | 1 | Heme | FE | 63.33 | 1 |
| | NA | 49.24 | | | NA | 67.63 | |
| | NB | 48.40 | | | NB | 67.26 | |
| | NC | 52.48 | | | NC | 67.97 | |
| | ND | 53.55 | | | ND | 66.10 | |
| His94 (F8) | NE2 | 52.06 | | His94 (F8) | NE2 | 66.16 | |
| His62 (E7) | NE2 | 48.31 | | His62 (E7) | NE2 | 65.15 | |
| Phe46 (CD1) | CE1 | 50.66 | | Phe46 (CD1) | CE1 | 63.85 | |
| Val66 (E11) | CG2 | 47.49 | | Val66 (E11) | CG2 | 59.86 | |
| Phe32 (B10) | CZ | 47.49 | | Phe32 (B10) | CZ | 65.88 | |
| | *Average* | 50.09 | | | *Average* | 65.32 | |
| | *S.D.* | 2.20 | | | *S.D.* | 2.44 | |
| $O_2$ | O1 | 50.95 | 0.75 | $O_2$ | O1 | 66.48 | 0.40 |
| | O2 | 48.38 | | | O2 | 66.01 | |
| | | | | | | | |
| A2 | | | | A2 | | | |
| Residue | atom | *B*-factor | Occ. | Residue | atom | *B*-factor | Occ. |
| Heme | FE | 43.04 | 1 | Heme | FE | 53.25 | 1 |
| | NA | 46.78 | | | NA | 53.76 | |
| | NB | 43.69 | | | NB | 52.23 | |
| | NC | 47.01 | | | NC | 55.71 | |
| | ND | 43.61 | | | ND | 55.49 | |
| His94 (F8) | NE2 | 48.11 | | His94 (F8) | NE2 | 61.13 | |
| His62 (E7) | NE2 | 45.09 | | His62 (E7) | NE2 | 57.46 | |
| Phe46 (CD1) | CE1 | 46.50 | | Phe46 (CD1) | CE1 | 56.91 | |
| Val66 (E11) | CG2 | 41.54 | | Val66 (E11) | CG2 | 53.91 | |
| Trp32 (B10) | CH2 | 48.11 | | Trp32 (B10) | CH2 | 57.47 | |
| | *Average* | 45.35 | | | *Average* | 55.72 | |
| | *S.D.* | 2.29 | | | *S.D.* | 2.64 | |
| $O_2$ | O1 | 46.13 | 0.65 | $O_2$ | O1 | 56.18 | 0.60 |
| | O2 | 44.28 | | | O2 | 56.16 | |
| | | | | | | | |
| B1 | | | | B1 | | | |
| Residue | atom | *B*-factor | Occ. | Residue | atom | *B*-factor | Occ. |
| Heme | FE | 47.20 | 1 | Heme | FE | 67.66 | 1 |
| | NA | 47.23 | | | NA | 66.28 | |
| | NB | 46.40 | | | NB | 63.17 | |
| | NC | 49.14 | | | NC | 68.13 | |
| | ND | 45.62 | | | ND | 67.12 | |
| His96(F8) | NE2 | 52.37 | | His96(F8) | NE2 | 66.66 | |
| Gln64 (E7) | NE2 | 55.19 | | Gln64 (E7) | NE2 | 66.41 | |
| Phe51 (CD1) | CE2 | 53.97 | | Phe51 (CD1) | CE2 | 64.17 | |
| Val68 (E11) | CG2 | 48.70 | | Val68 (E11) | CG2 | 61.46 | |
| Phe36 (B10) | CZ | 51.44 | | Phe36 (B10) | CZ | 66.57 | |

| | | | | | | | |
|---|---|---|---|---|---|---|---|
| | *Average* | 49.73 | | | *Average* | 65.76 | |
| | *S.D.* | 3.33 | | | *S.D.* | 2.13 | |
| $O_2$ | O1 | 49.62 | 0.60 | $O_2$ | O1 | 65.45 | 0.32 |
| | O2 | 49.74 | | | O2 | 66.78 | |
| B2 | | | | B2 | | | |
| Residue | atom | *B*-factor | Occ. | Residue | atom | *B*-factor | Occ. |
| Heme | FE | 48.85 | 1 | Heme | FE | 67.41 | 1 |
| | NA | 50.72 | | | NA | 71.23 | |
| | NB | 51.71 | | | NB | 72.04 | |
| | NC | 50.63 | | | NC | 72.60 | |
| | ND | 53.00 | | | ND | 74.11 | |
| His98 (F8) | NE2 | 45.55 | | His98 (F8) | NE2 | 66.70 | |
| His66 (E7) | NE2 | 52.02 | | His66 (E7) | NE2 | 64.49 | |
| Phe50 (CD1) | CE2 | 51.47 | | Phe50 (CD1) | CE2 | 69.37 | |
| Val70 (E11) | CG2 | 49.04 | | Val70 (E11) | CG2 | 66.30 | |
| Phe36 (B10) | CZ | 50.90 | | Phe36 (B10) | CZ | 61.95 | |
| | *Average* | 50.39 | | | *Average* | 68.62 | |
| | *S.D.* | 2.12 | | | *S.D.* | 3.90 | |
| $O_2$ | O1 | 49.30 | 0.70 | $O_2$ | O1 | 71.55 | 0.70 |
| | O2 | 48.94 | | | O2 | 71.84 | |

| **Cryst 3 (21% oxy)** | | | | **Cryst 4 (13% oxy)** | | | |
|---|---|---|---|---|---|---|---|
| A1 | | | | A1 | | | |
| Residue | atom | *B*-factor | Occ. | Residue | atom | *B*-factor | Occ. |
| Heme | FE | 36.14 | 1 | Heme | FE | 35.49 | 1 |
| | NA | 38.85 | | | NA | 36.97 | |
| | NB | 39.91 | | | NB | 38.46 | |
| | NC | 40.96 | | | NC | 38.62 | |
| | ND | 40.60 | | | ND | 38.55 | |
| His94 (F8) | NE2 | 42.98 | | His94 (F8) | NE2 | 37.43 | |
| His62 (E7) | NE2 | 32.97 | | His62 (E7) | NE2 | 38.44 | |
| Phe46 (CD1) | CE1 | 36.43 | | Phe46 (CD1) | CE1 | 40.18 | |
| Val66 (E11) | CG2 | 29.32 | | Val66 (E11) | CG2 | 27.36 | |
| Phe32 (B10) | CZ | 37.06 | | Phe32 (B10) | CZ | 34.88 | |
| | *Average* | 37.52 | | | *Average* | 36.64 | |
| | *S.D.* | 4.08 | | | *S.D.* | 3.62 | |
| $O_2$ | O1 | 38.11 | 0.25 | $O_2$ | O1 | 35.74 | 0.04 |
| | O2 | 40.01 | | | O2 | 35.41 | |
| A2 | | | | A2 | | | |
| Residue | atom | *B*-factor | Occ. | Residue | atom | *B*-factor | Occ. |
| Heme | FE | 38.10 | 1 | Heme | FE | 32.69 | 1 |
| | NA | 34.86 | | | NA | 33.57 | |
| | NB | 35.38 | | | NB | 32.37 | |
| | NC | 39.13 | | | NC | 34.24 | |
| | ND | 35.70 | | | ND | 35.46 | |
| His94 (F8) | NE2 | 39.17 | | His94 (F8) | NE2 | 34.54 | |
| His62 (E7) | NE2 | 38.36 | | His62 (E7) | NE2 | 28.69 | |

| | | | | | | | |
|---|---|---|---|---|---|---|---|
| Phe46 (CD1) | CE1 | 39.52 | | Phe46 (CD1) | CE1 | 28.23 | |
| Val66 (E11) | CG2 | 31.97 | | Val66 (E11) | CG2 | 28.70 | |
| Trp32 (B10) | CH2 | 37.03 | | Trp32 (B10) | CH2 | 31.71 | |
| | *Average* | 36.92 | | | *Average* | 32.02 | |
| | *S.D.* | 2.42 | | | *S.D.* | 2.64 | |
| $O_2$ | O1 | 37.02 | 0.05 | $O_2$ | O1 | 31.74 | 0.06 |
| | O2 | 37.25 | | | O2 | 31.21 | |
| | | | | | | | |
| B1 | | | | B1 | | | |
| Residue | atom | *B*-factor | Occ. | Residue | atom | *B*-factor | Occ. |
| Heme | FE | 38.88 | 1 | Heme | FE | 36.72 | 1 |
| | NA | 40.92 | | | NA | 38.48 | |
| | NB | 39.03 | | | NB | 34.65 | |
| | NC | 40.51 | | | NC | 38.28 | |
| | ND | 44.89 | | | ND | 36.60 | |
| His96(F8) | NE2 | 40.36 | | His96(F8) | NE2 | 39.45 | |
| Gln64 (E7) | NE2 | 45.34 | | Gln64 (E7) | NE2 | 40.71 | |
| Phe51 (CD1) | CE2 | 45.22 | | Phe51 (CD1) | CE2 | 45.01 | |
| Val68 (E11) | CG2 | 36.58 | | Val68 (E11) | CG2 | 38.56 | |
| Phe36 (B10) | CZ | 40.18 | | Phe36 (B10) | CZ | 39.46 | |
| | *Average* | 41.19 | | | *Average* | 38.79 | |
| | *S.D.* | 2.99 | | | *S.D.* | 2.79 | |
| $O_2$ | O1 | 41.94 | 0.15 | $O_2$ | O1 | 38.42 | 0.05 |
| | O2 | 42.75 | | | O2 | 38.40 | |
| | | | | | | | |
| B2 | | | | B2 | | | |
| Residue | atom | *B*-factor | Occ. | Residue | atom | *B*-factor | Occ. |
| Heme | FE | 47.30 | 1 | Heme | FE | 38.17 | 1 |
| | NA | 50.65 | | | NA | 42.05 | |
| | NB | 53.39 | | | NB | 43.15 | |
| | NC | 40.34 | | | NC | 41.89 | |
| | ND | 53.63 | | | ND | 41.18 | |
| His98 (F8) | NE2 | 40.34 | | His98 (F8) | NE2 | 34.94 | |
| His66 (E7) | NE2 | 44.06 | | His66 (E7) | NE2 | 34.71 | |
| Phe50 (CD1) | CE2 | 54.18 | | Phe50 (CD1) | CE2 | 41.95 | |
| Val70 (E11) | CG2 | 37.87 | | Val70 (E11) | CG2 | 34.53 | |
| Phe36 (B10) | CZ | 36.71 | | Phe36 (B10) | CZ | 32.42 | |
| | *Average* | 47.06 | | | *Average* | 38.50 | |
| | *S.D.* | 6.85 | | | *S.D.* | 4.01 | |
| $O_2$ | O1 | 50.92 | 0.30 | $O_2$ | O1 | 39.43 | 0.15 |
| | O2 | 51.49 | | | O2 | 39.29 | |

Occ.; Occupancy

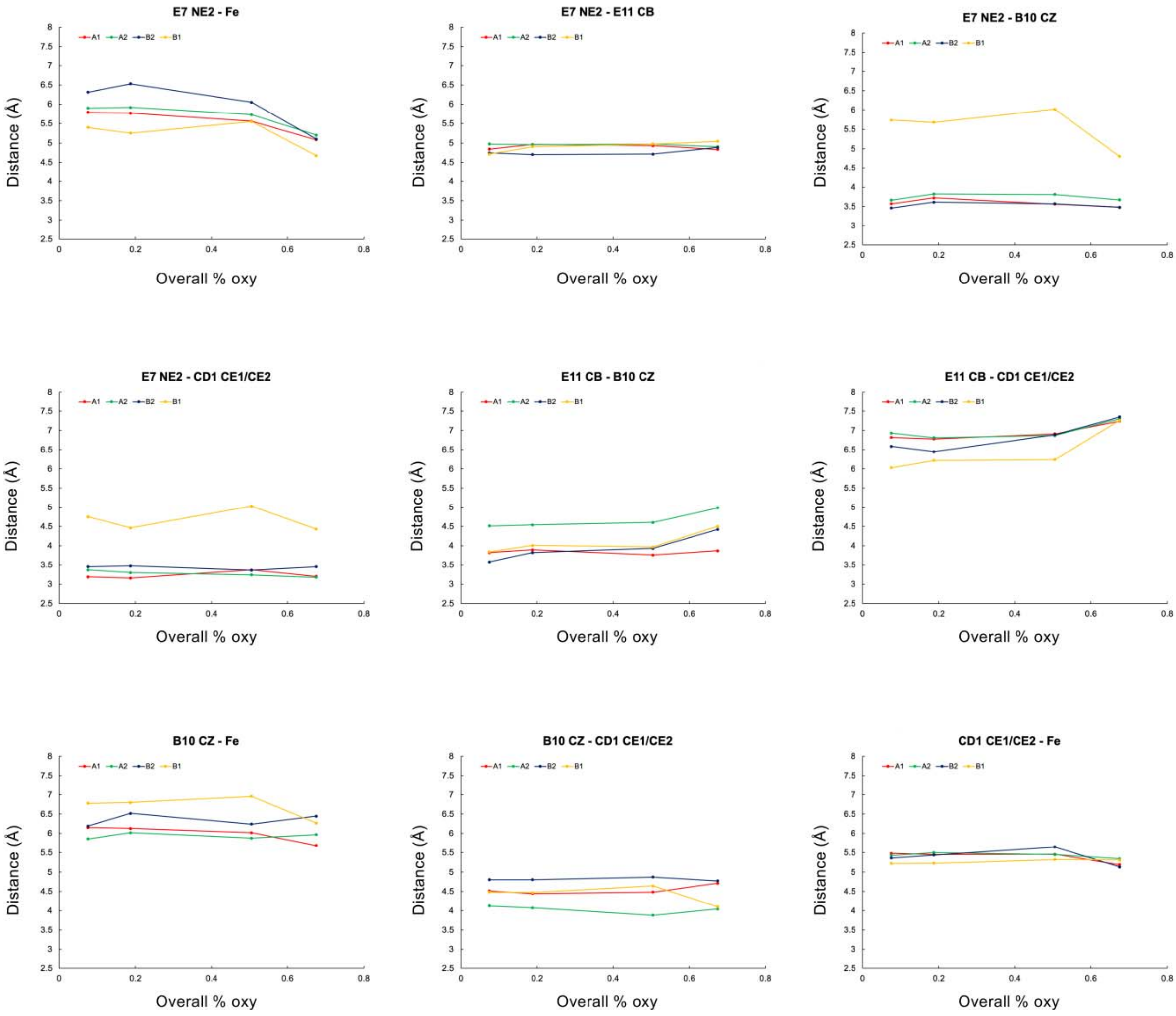


**Figure S1** Changes of the distance between the atoms of oxygen-surrounding residues for each subunit (A1, A2, B1, and B2 are drawn as red, green, blue, and yellow line, respectively). The combination of atoms whose distance was measured is shown in the upper part of each graph.
